# Disability and Happiness in Iran: What Can We Do?

**Published:** 2018-08

**Authors:** Shahin SOLTANI, Amirhossein TAKIAN

**Affiliations:** 1. Dept. of Health Management and Economics, School of Public Health, Tehran University of Medical Sciences, Tehran, Iran; 2. Dept. of Global Health and Public Policy, School of Public Health, Tehran University of Medical Sciences, Tehran, Iran; 3. Health Equity Research Center, Tehran University of Medical Sciences, Tehran, Iran

## Dear Editor-in-Chief

People with disability (PWD) experience poorer health than general population. The prevalence of mental disorders, i.e. depression, is higher among PWD than general population (1, 2). Depression is a major barrier to adapt to disability and new conditions in many PWD and their families (3, 4). Emotional and social isolation can aggregate secondary conditions and mental disorders in PWD (5–7).

The prevalence of depression is around 5.6% to 73% among various groups of population in Iran (1). The rate is even higher in PWD and elderlies, e.g., the prevalence of depression among elderlies who lived in their homes was about 85.81% (2).

PWD constitute 1–4 percent of general population in Iran (8), whose various life needs i.e. health, education, employment, entertainment, and leisure need special attention for moving the barriers to use such services. Financial, information and physical access are known as the most important factors to use services in different studies (9, 10). In addition, the complicated association between poverty and disability has led PWD to experience low participation in their communities.

To remedy this situation and improve PWD’s access to health and welfare services, an immediate national action plan is urgently required. Beyond political commitment to support and endorse the plan, the plan should, we propose, consist of:

- Facilitating financial access to medical, rehabilitation and physiological services through tailored health insurance schemes and provision of targeted subsidies;
- Considering special transportation and accommodation services to enhance access to health services for PWD;
- Paying particular attention to customized employment and educational opportunities for PWD;
- Enhancing public knowledge and understanding about disability within communities, through active engagament and policy dialogues with, but not limited to, families of PWD, media, schools, cultural and religious institutions;
- Conducting studies to identify the risk factors of depression among PWD, aiming to formulate policies for preventing depression.
- Implementation of screening programs for early detection of depression among high-risk individuals.

Lack of happiness is a common challenge among PWD. Health and welfare policymakers must boost their efforts, through evidence-based, immediate and tailored action plans, to identify the risk factors of depression, and plan for effective prevention and treatment interventions that are suitable in Iran. To fulfil this mission, it is crucial to consider appropriate financial, cultural and physical access to needed services at all levels.
